# Falls and falls-related injuries in individuals with chronic ankle symptoms: a cross-sectional study

**DOI:** 10.1186/s13047-023-00649-5

**Published:** 2023-08-16

**Authors:** Munira M. Al Mahrouqi, Bill Vicenzino, David A. MacDonald, Michelle D. Smith

**Affiliations:** 1 The University of Queensland, School of Health and Rehabilitation Sciences, Physiotherapy, Brisbane, QLD 4072 Australia; 2 Division of Physiotherapy, Oman College of Health Sciences, Ministry of Health, P.O. Box 3720, Muscat, PC 112 Sultanate of Oman; 3 Physiotherapy, School of Health Sciences and Social Work, Griffith University, Gold Coast, QLD 4222 Australia

**Keywords:** Ankle pain, Ankle osteoarthritis, Falls, Falls self-efficacy

## Abstract

**Background:**

Falls are a major public health concern globally. While falls are associated with osteoarthritis and persistent pain at the hip and knee, falls have not been investigated in people with chronic ankle symptoms. This study aimed to compare self-reported history of falls between adults with and without chronic ankle symptoms. Secondary aims were to compare concern about falling and balance confidence between groups, and to identify factors associated with falling.

**Methods:**

A total of 226 participants (134 with chronic ankle pain and/or stiffness and 92 controls) participated in this cross-sectional case–control study. Participants completed an online questionnaire about falls in the past 12 months, injuries associated with falling, concern about falling, balance confidence, function, pain and multimorbidity.

**Results:**

Eighty-six (64%) participants with chronic ankle symptoms and 24 (26%) controls reported at least one fall in the last 12 months (*p* < 0.001). Participants with chronic ankle symptoms reported more falls, more injurious falls, and more hospitalisations because of a fall than controls (*p* > 0.002). There was a small effect for lower balance confidence and higher concern about falling in symptomatic participants (standardised mean difference: 0.39–0.49; *p* > 0.017). Logistic regression analysis identified that falling was associated with the presence of ankle symptoms (3.08 (1.20, 7.92); *p* = 0.02) and concern about falling (odds ratio (95% confidence intervals): 1.13 (1.05, 1.23); *p* = 0.002).

**Conclusions:**

Falls and falls-related injuries are a problem in individuals with chronic ankle symptoms. The high falls occurrence and concern about falling in individuals with chronic ankle symptoms suggest the need for clinicians to assess these factors in this population.

**Supplementary Information:**

The online version contains supplementary material available at 10.1186/s13047-023-00649-5.

## Background

Falls are a significant health concern that can result in serious injury, functional decline, loss of independence, hospitalization and even death [1, 2]. Although falls occur across the lifespan, they are more common among older adults, with one in three adults 65 years of age and older falling annually [3]. In older adults, chronic joint pain is associated with an increased risk of falls [4] and greater fear of falling [5]. Persistent knee [6] and hip [7] pain have specifically been identified as falls risk factors, but incidence of falls has not been investigated in people with persistent ankle pain despite this being a prevalent problem [8]. Qualitative research on individuals with ankle osteoarthritis has identified that this population has concerns about falling and reports instability as a key symptom of their condition [9]. This suggests that falls may be a problem in this population.

Many factors, such as impaired balance [10] and ankle muscle weakness [11], have been suggested to increase falls risk. Individuals with ankle osteoarthritis and chronic ankle symptoms have impaired balance [12, 13], decreased muscle strength [14, 15], reduced ankle joint motion [14, 15] and decreased mobility [15]. In light of the relationship between these impairments and falls, individuals with ankle osteoarthritis and chronic ankle symptoms may be at an increased risk of falling. Further, chronic ankle symptoms, including osteoarthritis, are a consequence of ankle sprains and fractures, which are associated with mechanoreceptor damage and compromised proprioception [16], ankle instability and giving way [15, 17]. These impairments may impair balance and increase risk of falls.

In order to better understand whether falls are a problem in individuals with chronic ankle symptoms, the primary aim of this study was to compare self-reported history of falls between adults with and without chronic ankle symptoms. Secondary aims were to compare concern about falling and balance confidence between groups, and to identify factors associated with falling.

## Methods

A cross-sectional online questionnaire compared history of falls, concern about falling and balance confidence between adults with and without chronic ankle symptoms. The study was approved by the institutional Human Research Ethics Committee (Approval #: 2014001194) and all participants provided informed consent. The study is reported in accordance with the STROBE checklist (Supplementary file 1) [18].

### Participants

A convenience sample of individuals living in Australia with and without chronic ankle symptoms were recruited via community advertisements, national and state arthritis organization websites, and social media between March 2017 and April 2018. Individuals were informed that the study would help understand problems experienced by people with ankle pain, and that we were looking for people with persistent ankle pain and people without ankle pain (to act as comparators) to participate. Adults with chronic ankle symptoms (symptomatic group) were eligible for inclusion if they experienced ankle joint pain rated as ≥ 2 out of 10 on an 11-point numerical rating scale (NRS) anchored with the ‘No pain’ at 0 and ‘Worst pain imaginable’ at 10 [19], and/or stiffness or reduced movement of ankle in the morning on most days for > 3 months. Participants who had pain in other body regions that was equal to or greater than their ankle pain were excluded. The inclusion criteria for participants with chronic ankle symptoms was based on previous research that found 94% of individuals who have ankle joint pain and/or stiffness for > 3 months have radiographic ankle osteoarthritis [15]. Participants without chronic ankle symptoms were required to have no history of ankle injury, and no ankle pain or stiffness in the past 3 months. Eligibility criteria was assessed via an eligibility questionnaire. Eligible individuals who were interested in participating in the study were provided a link to the online questionnaire.

### Questionnaire

Data was collected using an online platform (SurveyMonkey®). Participants were asked about their history of falling within the last year, concern about falling and balance confidence. They also provided information on their age, sex, function, comorbid health conditions, ankle pain severity, and pain in bodily locations. The questionnaire was developed by the research team and piloted for functionality and readability prior to data collection. No incentives were offered for study completion. Questions were presented in a standardised order with embedded logic to display follow-up questions as relevant to previous responses (e.g., if a participant answered ‘yes’ to suffering an injury from a fall, they were asked subsequent questions about injuries sustained).

### Outcome measures

Falls in the last 12 months was determined by the question: “In the last 12 months, have you had any falls?” A fall was defined as ‘an event which results in a person coming to rest inadvertently on the ground, floor or other lower level’ [20]. Participants were categorised as fallers (an individual who fell at least once over the last 12 months) or non-fallers. Participants indicated the number of falls they experienced in the past 12 months from options ranging from ‘0’ to ‘5 or more’. Individuals who selected ‘5 or more’ falls were attributed 5 falls for calculation of the total number of falls. Fallers indicated if they sustained an injury from falling, the type of injury experienced (i.e., bruises, cuts/grazes, sprain/strain, broken bones, dislocation), and if any fall resulted in hospitalization.

The Falls Efficacy Scale-International (FES-I) was used to measure concern about the possibility of falling when performing 16 different physical and social activities [21]. Level of concern was measured on a Likert scale ranging from 1 (‘not at all concerned’) to 4 (‘very concerned’). The total (summed) score ranged from 16 to 64, with a higher score indicating a greater concern about falling (i.e., lower falls self-efficacy). Concern about falling was categorised as: low (scores of 16–19), moderate (scores of 20–27) and high (scores of 28–64) [22]. This instrument has excellent internal and test–retest reliability [21].

The Activities-Specific Balance Confidence (ABC) scale, which has established reliability, was used to measure balance confidence during activities of daily living [23]. Participants were asked to indicate their level of confidence performing 16 activities without losing balance or becoming unsteady. Confidence was rated on a scale from 0% (‘not confident at all’) to 100% (‘completely confident’) with answers provided in 10% increments. An average score was calculated. Scores ranged from 0 to 100%, with higher scores indicating better balance confidence.

The 21-item Activities of Daily Living subscale of the Foot and Ankle Ability Measure (FAAM-ADL) was used to assess function [24]. Each item was scored on a 5-point Likert scale ranging from ‘no difficulty’ (4) to ‘unable to do’ (0). The total score (sum of responses) was converted to a percentage (0–100%), with a higher percentage indicating a higher level of function. This questionnaire has excellent test–retest reliability and internal consistency [24].

Severity of ankle pain was measured using an 11-point NRS with 0 anchored with “no pain” and 10 anchored with “worst pain imaginable”. Participants were asked to indicate the worst ankle pain experienced during the past 7 days. This measure has excellent reliability [25]. Pain in 12 other bodily regions (neck, shoulders, upper back, elbows, wrists/hands, low back, right hip/thigh, left hip/thigh, right knee, left knee, right foot and left foot—identified on a body chart) was also recorded using an NRS [26]. The number of bodily regions (including the ankle) with pain ≥ 2 out of 10 were summed as a measure of the number of bodily pain sites (score ranging from 0 to 13).

A modified version of the Self-Administered Comorbidity Questionnaire was used to collect data on multimorbidity [27]. Participants indicated if they experienced and received treatment for the following chronic health problems: heart disease, high blood pressure, lung disease, diabetes, ulcer or stomach disease, kidney disease, liver disease, anaemia or other blood disease, cancer, depression, back pain, auto-immune disease, rheumatoid arthritis, gouty arthritis, and osteoarthritis at joints other than ankle. There was also option for participants to identify any ‘other’ health conditions not listed. Multimorbidity was defined as the presence of two or more chronic health conditions (yes/no) [28, 29].

### Data and statistical analysis

Questionnaire data was reviewed for completion and participants who did not complete the questions on falls history, concern about falling (FES-I) and balance confidence (ABC scale) were excluded from analysis. Statistical analyses were performed using IBM SPSS Statistics for Windows (Version 25.0. Armonk, NY: IBM Corp).

Chi-square tests were conducted to compare categorical and binary variables (sex, falls status, number of falls, falls-related injuries, falls-related hospitalisation, concern about falling (FES-I categories) and multimorbidity) between participants with chronic ankle problems and controls. A univariate analysis of covariance (ANCOVA) was used to compare differences in concern about falling, balance confidence, and ADL function between participants with and without chronic ankle symptoms. Age, sex, and severity of pain in body areas other than the ankle were included as covariates. Age was compared between groups using an independent t-test. As data for number of bodily pain sites and severity of pain were not normally distributed, Wilcoxon rank-sum tests were used to compare these outcomes between groups.

Categorical and binary variables are presented as frequency (percentage), and normally and non-normally distributed continuous variables are presented as mean (standard deviation (SD)) and median (inter-quartile range (IQR)), respectively. For comparison of falls outcomes between groups, risk differences (95% confidence intervals (CI)) were calculated for categorical/binary variables and standardized mean differences (SMD; 95% CI) were calculated for continuous variables. Effect sizes were interpreted as trivial: 0.0–0.2, small: 0.2–0.6, medium: 0.6–1.2, large: 1.2–2.0, very large: 2.0–4.0 and distinct: > 4.0 [30].

The relationship between falls status (dependent variable) and independent variables (age, sex, concern about falling, balance confidence, ADL function, ankle pain severity, pain severity excluding the ankle, number of pain sites, multimorbidity, depression (a binary outcome from the Self-Administered Comorbidity Questionnaire), and group (entered into the model through a dummy coded variable)) was investigated using logistic regression. Variables significant at *p* < 0.1 were included in multivariate analysis. A backward elimination regression model was conducted to investigate independent variables associated with falls status (i.e., faller vs non-faller). The model was tested for multicollinearity. Variables were sequentially eliminated leaving only those with *p* < 0.1. Logistic regression data are reported as odds ratios and 95% CI.

## Results

### Participant demographic and health outcomes

A total of 277 individuals were assessed for eligibility with 20 individuals excluded due to other areas of pain more severe than that at the ankle (*n* = 17), individuals in the control group with ankle pain (*n* = 2) and age < 18 years (*n* = 1), leaving 257 individuals who participated in the questionnaire (93% of those who were eligible). Complete data was available for 226 participants who commenced the questionnaire (88% completion rate). There were 134 participants with chronic ankle symptoms and 92 controls (152 females and 74 males; mean (SD) age of 54.3 (12.9) years). The median (IQR) for ankle pain intensity for the symptomatic group was 7 (5–9), with no ankle pain in controls. Participants with and without chronic ankle symptoms were similar in age and sex (all *p* > 0.87), but those with chronic ankle symptoms had poorer ADL function, a higher number of musculoskeletal pain sites, greater pain severity at sites other than the ankle, and were more likely to have multimorbidity (all *p* < 0.001; Table 1).

**Table 1:** Demographic outcomes for participants with chronic ankle symptoms (*n* = 134) and controls (*n* = 92)

	**Chronic ankle symptoms**	**Controls**	***p*-value**
Female, n (%) ^a^	91 (68%)	61 (66%)	0.80
Age, years ^b^	54.2 (12.3)	54.5 (13.7)	0.87
FAAM-ADL, % ^c^	67.1 (14.9)	91.5 (15.4)	< 0.001
Number of musculoskeletal pain sites, /13 ^d^	7 (4, 10)	1 (0, 3)	< 0.001
Pain severity at sites other than the ankle, /10 ^d^	6 (3, 8)	2 (0, 3)	< 0.001
Multimorbidity, n (%) ^a^	68 (51%)	20 (22%)	< 0.001

*FAAM-ADL *The Foot and Ankle Ability Measure-Activities of Daily Living subscale

FAAM data are based on 51 control and 69 symptomatic due to missing data

^a^Data presented as number (%) and analysed with chi-squared tests

Data presented as mean (standard deviation) and analysed using ^b^ t-tests and ^c^ ANCOVA (age, sex, and severity of pain in body in areas other than the ankle as covariates)

^d^Data presented as median (inter-quartile range) and analysed using Wilcoxon rank-sum tests

### Falls outcomes

A total of 220 falls were reported by study participants. Nearly half (49%; *n* = 110) of all participants reported at least one fall in the last 12 months, with 27% of participants (*n* = 60) reporting more than one fall. Among fallers, 41% of participants sustained a fall-related injury. The most reported injury type was bruises, cuts and grazes (46%).

There were 186 falls reported among participants with chronic ankle symptoms and 34 falls reported by controls. There were significantly more fallers (individuals with one or more falls; *p* < 0.001), more participants who reported multiple (> 1) falls (*p* = 0.005), and more participants who sustained an injury from falling (*p* = 0.002) in the chronic ankle symptoms group than the control group (Table 2). All injury types, including serious injuries (e.g., fractures/dislocations) and hospitalisations, were more common in fallers with ankle symptoms than fallers in the control group (Table 2).

**Table 2:** Falls-related outcomes for participants with chronic ankle symptoms (*n* = 134) and controls (*n* = 92)

	**Chronic ankle symptoms**	**Controls**	**Effect size**
Fallers, n (%)	86 (64%)	24 (26%)	38% [26, 50]
Fallers with multiple (> 1) falls, n (%)	53 (40%)	7 (8%)	0.3% [0.2, 0.4]
Injured fallers, n (%) ^b^	41 (31%)	3 (3%)	35% [18, 52]
Bruises/cuts/grazes, n (%) ^b^	47 (55%)	3 (13%)	42% [25, 59]
Sprains/strains, n (%) ^b^	16 (19%)	1 (4%)	14% [3, 26]
Fractures/dislocations, n (%) ^b^	14 (16%)	0 (0%)	16% [7, 26]
Hospitalisations, n (%) ^b^	13 (32%)	0 (0%)	32% [4, 67] ^
High concern about falling, n (%)	44 (33%)	1 (1%)	32% [24, 40]
Moderate concern about falling, n (%)	52 (39%)	12 (13%)	26% [15, 37]
Low concern about falling, n (%)	38 (28%)	79 (86%)	-58% [-68, -47]
FES-I, 16–64^a^	24.3 (7.9)	21.4 (8.2)	2.9 [0.5, 5.2]
ABC, %^a^	78.4 (19.9)	88.4 (20.7)	-10.0 [-15.9, -4.1]

*ABC *The Activities-Specific Balance Confidence Scale, *FES-I *The Falls Efficacy Scale-International

Data are presented as number (%) and risk difference (RD) (95% CI), and analysed using chi-squared tests unless otherwise stated

^a^Data presented as mean (standard deviation) and mean differences (MD) and 95% confidence interval (CI), and analysed using ANCOVA (age, sex, and severity of pain in body in areas other than the ankle as covariates)

^b^Percentage is calculated from the number of fallers in each group (e.g., # hospitalized/# fallers)

Participants with chronic ankle symptoms were 32% more likely to report high concern about falling, and 26% more likely to report moderate concern, than those in the control group (Table 1). There were small effect sizes for higher concern about falling (FES-I; SMD (95% CI) = 0.4 (0.1, 0.6; *p* = 0.017) and lower balance confidence lower balance confidence (ABC scale; SMD (95% CI) = 0.5 (0.8, 0.2); *p* = 0.001) in participants with ankle symptoms compared to controls (Table 2).

### Outcomes associated with falls status

Univariate associations between falls status (dependent variable; being a faller or non-faller) and independent variables balance confidence (ABC Scale), concern about falling (FES-I), group (chronic ankle symptoms or control), function (FAAM-ADL), ankle pain severity (NRS), number of pain sites, pain severity at sites excluding the ankle (NRS), multimorbidity, depression, age, and sex are reported in Table 3. As sex and age were not significantly associated with falls status in univariate analysis at *p* < 0.1, they were not included in the multivariate regression model. All other variables were included in the regression model with each variable removed sequentially leaving only concern about falling and group associated with falls status (R^2^ = 0.235; Table 4). Fallers had greater concern about falling and were more likely to have chronic ankle symptoms than non-fallers.

**Table 3:** Univariate associations between independent variables and falls status (faller vs non-faller)

Independent variables	OR (95% CI)	*p*-value
Group	5.08 (1.83, 9.10)	< 0.001
FES-I	1.15 (1.09, 1.22)	< 0.001
Multimorbidity	2.32 (1.35, 4.02)	0.003
ABC scale	0.96 (0.94, 0.97)	< 0.001
Number of pain sites	1.23 (1.14, 1.32)	< 0.001
FAAM-ADL	0.96 (0.94, 0.98)	< 0.001
Ankle pain severity	1.26 (1.17, 1.37)	< 0.001
Pain excluding ankle	1.33 (1.20, 1.47)	< 0.001
Depression	1.99 (1.02, 3.87)	0.04
Age	1.01 (0.99, 1.03)	0.49
Sex	0.85 (0.49, 1.49)	0.57

*OR* Odds ratios, *CI* Confidence intervals, *ABC* Activities-Specific Balance Confidence Scale, *FES-I* Falls Efficacy Scale-International, *FAAM-ADL* The Foot and Ankle Ability Measure-Activities of Daily Living Subscale

All data based on full sample *n* = 226, except FAAM ADL *n* = 120

Logistic regression analysis

Group (chronic ankle symptoms or control)

**Table 4:** Variables retained in the final multivariate logistic regression model and their associations with falls status (faller vs non-faller)

Variables	OR (95% CI)	*p*-value
Group	3.08 (1.20, 7.92)	0.02
FES-I	1.13 (1.05, 1.23)	0.002

*OR *Odds ratios, *CI* Confidence intervals, *FES-I* Falls Efficacy Scale-International

Group (chronic ankle symptoms or control)

## Discussion

The primary aim of this study was to compare self-reported history of falls between individuals with and without chronic ankle symptoms. Our data indicate that falls, and becoming injured or hospitalised because of a fall, are more prevalent in individuals with chronic ankle symptoms than those without ankle symptoms. With nearly half of fallers with chronic ankle symptoms suffering an injury and nearly one third requiring hospitalisation, our findings highlight the seriousness of falling in this population. Secondary aims of this study were to compare concern about falling and balance confidence between individuals with and without chronic ankle symptoms, and to identify factors associated with falling. Individuals with chronic ankle symptoms had a greater concern about falling (FES-I) and lower balance confidence than controls, which are characteristics linked to an increased risk of falls in other populations [31]. Among our participants, falls status (being a faller or non-faller) was related to concern about falling and whether an individual had chronic ankle symptoms (compared to no ankle symptoms). These data suggest the need to address falls and concern about falling in individuals with chronic ankle symptoms.

Impairments in muscle strength, ankle range of motion, balance and ambulatory function have been identified in individuals with chronic ankle symptoms [14, 15]. As these characteristics have been linked to falls in other populations [10, 11], it is possible that they may contribute to an increased risk of falling in individuals with chronic ankle symptoms. Our data suggest that individuals with chronic ankle symptoms are more likely to experience multimorbidity than individuals without ankle concerns. Many health conditions reported by our participants with ankle symptoms, such as back pain [32] and depression [33], are associated with an increased risk of falls. Research has identified a relationship between the number of falls risk factors an individual possesses and increased risk of falling [34]. Thus, multiple risk factors identified in individuals with chronic ankle symptoms may contribute to the increased falls in this population.

A high proportion of individuals with chronic ankle symptoms in our study (72%) had high to moderate concerns about falling. This finding supports data from qualitative work which identified concerns about falling in people with ankle osteoarthritis [9]. Ankle instability, a common complaint in people with chronic ankle symptoms [35] and ankle osteoarthritis [15], and low balance confidence identified in our study, have been linked to concerns about falling in previous work [9]. Future research should investigate the effect of addressing these factors and other falls risk factors on falls incidence and concerns in people with chronic ankle symptoms.

As most research on falls has focused on older adults it is difficult to compare our falls data (from individuals with a mean age of 54 years) to that from other studies. While almost half of our study participants were fallers, only 26% of individuals in our control group fell in the last 12 months. Our control data is similar to that of a previous study which reported a 12-month falls rate of 20% in individuals aged 20–87 years [36]. Other studies have reported that 55% of older adults with chronic musculoskeletal pain experienced a fall in an 18-month period [4] and 59% of individuals with rheumatoid arthritis experienced a fall in a 12-month period [37]. This is similar to our finding that 64% of participants with chronic ankle symptoms experienced a fall in the previous year.

The population investigated in this study were individuals who had ankle joint pain during weight-bearing activities and/or stiffness for at least three months. While we did not take radiographs of this sample, we expect that many individuals in this study would have ankle osteoarthritis. This is based on similarities in the characteristics of our sample to the National Institute for Health and Care Excellence guidelines for the diagnosis of osteoarthritis [38], and research showing that 94% of people with chronic ankle joint pain and stiffness have radiographic ankle osteoarthritis (Kellgren and Lawrence grade of ≥ 2 (osteophytes with mild-severe joint space narrowing) [15].

While this the first study to investigate falls in individuals with chronic ankle symptoms and highlights the seriousness of falls in this population, there are limitations to consider. First, the study was cross-sectional and relied on recall to report falls, number of falls and falls-related injuries experienced in the past 12 months. While we hypothesise that chronic ankle symptoms increase the risk of falling, we cannot conclusively rule out that falling did not lead to the chronic ankle symptoms. Prospective cohort studies are needed to confirm our findings, ascertain causality and increase understanding of the circumstance surrounding falls in individuals with chronic ankle symptoms. Second, this study collected data via an online questionnaire. Because of this, participation was limited to internet users, and the FES-I and ABC scale questionnaires were administered online, rather than in their traditional paper format. Third, the questionnaire did not enquire about medication use which may be higher in individuals with chronic ankle pain and has been associated with falls [20]. Future research could consider the relationship between medication use and falls in this population.

Findings from this study suggest that healthcare professionals should ask patients presenting with chronic ankle symptoms and ankle osteoarthritis about their falls history and any concerns they have about falling. Clinicians may want to assess falls risk factors in these patients to identify impairments that can be targeted in management. Exercise management may be considered to address risk factors such as impaired balance [10] and ankle muscle weakness [11], that are common in people with chronic ankle problems [12–15]. Implementation of cognitive-behavioural therapies to reduce concern about falling and improve self-efficacy has been shown to be effective in reducing falls in other populations [39]. In light of high concerns about falling and the relationship between concerns about falling and having a fall, this may be beneficial for individuals with chronic ankle symptoms. Research on the effect of falls prevention interventions on falls incidence and concern about falling in individuals with chronic ankle symptoms and ankle osteoathritis is warranted.

## Conclusion

Falls and sustaining an injury from a fall are more prevalent in individuals with chronic ankle symptoms compared to individuals with no ankle symptoms. Concern about falling and ankle symptoms may be important to consider when managing patients with chronic ankle symptoms to decrease risk of falls in this population.

### Supplementary Information

**Additional file 1:** **Supplementary File 1.** STROBE Statement – Checklist of items that should be included in reportsof observational studies.

## Data Availability

The datasets generated and/or analysed during the current study are available in The University of Queensland eSpace and are available from the corresponding author on reasonable request.

## Abbreviations

NRS: Numerical rating scale
ABC: The Activities-Specific Balance Confidence Scale
FES-I: The Falls Efficacy Scale-International
FAAM-ADL: The Foot and Ankle Ability Measure-Activities of Daily Living subscale
RD: Risk difference
MD: Mean differences and CI = confidence interval
